# Intracellular invasion potential and pathogenic effects of *Corynebacterium striatum* clinical isolates in human airway epithelial cells

**DOI:** 10.3389/fmicb.2025.1647771

**Published:** 2025-07-28

**Authors:** Lanna Du, Binxin Guo, Juan Wen, Hui Liu, Junrui Wang

**Affiliations:** ^1^Department of Laboratory Medicine, Affiliated Hospital of Inner Mongolian Medical University, Hohhot, China; ^2^Department of Pediatrics, Affiliated Hospital of Inner Mongolian Medical University, Hohhot, China

**Keywords:** *Corynebacterium striatum*, intracellular invasion, human A549 epithelial cells, virulence-related genes, pathogenesis

## Abstract

**Background:**

*Corynebacterium striatum* emerged as an important hospital acquired pathogen in recent years, but less is known about its virulence potential. This study focusses on its pathogenesis on human airway epithelial cells, since lower airway tract infection was the most frequent type of infection caused by *C. striatum*.

**Methods:**

Whole genome sequencing was employed to construct single nucleotide polymorphism (SNP)-based phylogenetic tree of 27 *C. striatum* clinical isolates and predict the carriage of virulence related genes. Adherence and invasion capabilities of these isolates toward human A549 epithelial cells were detected using antibiotic protection assay, and the pathogenic effects of *C. striatum* to A549 cells was detected by flow cytometry.

**Results:**

Twenty-seven *C. striatum* clinical isolates were classified into five clades and 62.96% (17/27) isolates belonged to the predominant clade five, all of which carried seven virulence related genes (*hmuU, irp6B, regX3, groEL, sigA, sodA*, and *sigH*). Based on the protocol established for invasion assay in this study, 44.44, 48.15 and 7.41% isolates were classified as strongly invasive (SI), moderately invasive (MI), and weakly invasive (WI) isolates, respectively. All of the isolates could effectively invade into A549 cells during 2h infection, with varying invasion rates from 0.001% to 4.615%. The highest apoptosis rate (30.54%) was observed in A549 cells infected by the representative SI isolates (CS-51), followed by 25.56% for CS-252 (SI), 24.95% for CS-32 (MI), and 17.53% for CS-258 (MI).

**Conclusions:**

To our knowledge, this is the first report to characterize the *in vitro* intracellular invasion and pathogenesis of *C. striatum*. All of the *C. striatum* isolates tested in this study could effectively invade into A549 cells and the representative isolates displayed obvious cytotoxicity with varying degrees. The contribution and mechanism of specific virulence-related genes in mediating intracellular invasion in *C. striatum* needs further investigation, especially for *spaDEF*.

## 1 Introduction

Over the last few decades, multidrug resistant *C. striatum* has implicated in multiple invasive infections in immunosuppressed and immunocompetent patients worldwide, especially in lower respiratory tract and bloodstream infections (Ramos et al., 2019; Wang et al., 2021). One of the features of *C. striatum* is its biofilm production capability, which has been confirmed to be an important virulence determinant contributing to its nosocomial transmission and infection (Souza et al., 2015, 2020). However, no clear correlation between biofilm production ability and the virulence potential of *C. striatum* has yet been determined.

Bacterial pathogens with biofilm production abilities can adhere to multiple types of surfaces and host cells with the aid of specific bacterial surface structures, including pili and surface adhesins (Jacobsen et al., 2020; van Belkum et al., 2021). Pili encoded by *spa* gene clusters are universally distributed among *C. striatum* clinical isolates and are involved in adherence to host cells (Alibi et al., 2021). A similar phenomenon has also been observed among other *Corynebacterium* spp., such as *C. diphtheriae* and *C. urealyticum* (Guimarães et al., 2015; Sue et al., 2024). However, the actual role of these surface structures in mediating subsequent intracellular invasion and cytotoxicity remains unclear.

To the best of our knowledge, few reports have investigated the possible mechanisms underlying virulence potential of *C. striatum*. Alibi et al. (2021) reported that *C. striatum* could show virulence against *C. elegans*; however, the virulence potential was independent of the biofilm production capacity. Souza et al. (2019) found that all 22 tested *C. striatum* strains infected and killed *C. elegans* after 5 days of infection, and all of the isolates expressed *spaDEF* gene clusters and produced biofilms on several types of surfaces, which indicated a potential virulence mechanism mediated by *spaDEF*. In our previous study, the distribution of *spaDEF* gene clusters varied greatly among different *C. striatum* clinical isolates, and all *C. striatum* isolates with strong biofilm production capabilities expressed *spaDEF*, whereas the isolates with medium or weak biofilm production capabilities did not (Wen et al., 2022). No available report has validated the invasion capability of *C. striatum* isolates into human cells, and it is also not clear whether the invasion ability of *C. striatum* is accompanied by elevated intracellular proliferation ability. One report identified over 15 different virulence factors among *C. striatum* clinical isolates (Qiu et al., 2023), but the specific roles of these genes were not elucidated.

In this study, we aimed to ascertain the intracellular invasion and cytotoxicity potentials of *C. striatum* clinical isolates and to explore the characteristics of the carriage of virulence-related genes among *C. striatum* isolates with different intracellular invasion capabilities.

## 2 Methods

### 2.1 Bacterial isolates

Twenty-seven clinical *C. striatum* isolates were collected from a tertiary teaching hospital from the routine biological specimens, which were obtained during the clinical diagnosis and management of the patients. The detailed demographic information was described in our previous study (Wen et al., 2022). The isolates were subcultured on blood agar plates and incubated at 37°C for 24 h. Rights and health of the subjects was not under threat, and no personal identifying information was used during this study. Thus, this work is exempt from formal ethical approval and informed consent according to the local ethical guidelines, and was approved by the Ethics Committee of the Inner Mongolian Medical University (Reference Number: YKD202202033).

### 2.2 Whole-genome sequencing and prediction of virulence-related genes

The genomic DNA of *C. striatum* cultures was extracted using TIANamp Bacteria DNA Kit (Beijing Tiangen Company, China). Subsequently, 1 μg of total DNA was used as the input material for DNA sample preparation. Sequencing libraries were generated using a NEBNext^®^ Ultra™ DNA Library Prep Kit (Illumina, NEB, USA) according to the manufacturer's recommendations, and index codes were added to attribute sequences to each sample. The whole genomes of the 27 *C. striatum* isolates were sequenced using Illumina NovaSeq PE150 at Genekinder Medicaltech Co., Ltd. (Shanghai, China). Genome assembly with clean data was performed, and a single-nucleotide polymorphism (SNP)-based phylogenetic tree was constructed using TreeBeST and PhyML, with a bootstrap setting of 1,000 for orthologous genes. The whole-genome sequence of *C. striatum* NCTC9755 was used as the reference sequence (Genbank number, UFYO01000001.1). The Pathogen Host Interactions (PHI) (Urban et al., 2015) and Virulence Factors of Pathogenic Bacteria (VFDB) (Chen et al., 2012) databases were used to predict the functions of the genes. Results with an alignment coverage of ≥80% and alignment identity of ≥60% were predicted as virulence factors in this study, based on the standard employed by Qiu et al. (2023).

### 2.3 Adherence and invasion assay

The procedures for the determination of adherence and invasion were based on the protocol introduced by Ramos-Vivas et al. (2011) with some minor modifications. A total of 27 clinical isolates of *C. striatum* and one standard strain (BNCC 327370) were used in these assays. Human epithelial A549 cells were cultured in F-12K complete medium with 10% fetal bovine serum and 5% CO_2_ at 37°C and subcultured every 2–3 days. Cells were seeded into 24-well plates at a density of 1 × 10^5^ per well. *C. striatum* colonies were picked from blood agar plates and cultured in 5 mL of tryptic soybean broth at 220 rpm and 37°C until they reached the logarithmic growth phase. Bacterial concentration was determined by measuring absorbance at 620 nm and plotting it on a standard curve with known concentrations of CFU values. The culture was then diluted to the required concentration. A549 cells were inoculated at a multiplicity of infection (MOI) of 100 bacteria/cell. Infected cells were incubated for 2 h at 37°C in a 5% CO_2_ atmosphere to allow adhesion. The cells were then washed five times to remove the unbound bacteria, and 1% Triton X-100 was added to permeabilize the membranes of the cells. Cells were lysed in 1 mL of distilled water for 15 min and mechanically detached to obtain the lysates. After serial dilutions, 10 μL of the lysate was plated onto blood agar plates and incubated for 48 h at 37°C under 5% CO_2_. The average number of colonies was recorded, and the adhesion rate was calculated for each assay.

To investigate the invasive capability of the *C. striatum* strains in A549 cells, the following protocol was adopted. After 2 h of infection using the same procedure as that for the adherence assay, the unbound bacteria were removed by washing with PBS five times, and the cells were treated with gentamicin (200 μg/mL) for 1 h to eliminate extracellular bacteria. To detect the sensitivity of 27 clinical isolates of *C. striatum* to gentamicin (200 μg/mL), a standard broth microdilution method was employed during this study. To further confirm that gentamicin at 200 μg/mL could effectively eliminate extracellular bacteria, a control experiment was performed in triplicate, by which the supernatant from the wells with infected cells (prior to Triton X-100 lysis) was collected and plated onto blood agar plates. After incubation for 48 h at 37°C under 5% CO_2_, the colonies on plates were counted. To release intracellular bacteria, cells were lysed using the same method as that for adherence assay. The lysates were diluted for the detection of viable bacteria following the same procedure as that used for the adherence assay. The average number of colonies was recorded, and the invasion rate was calculated. All assays were performed in triplicate. To mitigate the impact of varying growth rates among different *C. striatum* strains on their ability to invade A549 cells, growth curves were plotted by measuring absorbance at 620 nm every 2 h over a 24-h period in a liquid shaking culture at 37°C under 5% CO_2_.

### 2.4 Immunofluorescence staining

A549 cells were seeded at a density of 2 × 10^5^ onto 12-mm diameter coverslips in 24-well plates. Then, an MOI of 10:1 (bacterium/A549 cell ratio) was used for *in vitro* infection. Following incubation at 37°C for 2h or 24 h, cells were washed four times and fixed with cold paraformaldehyde (3.2% in PBS) for 20 min at room temperature, as described previously (Ramos-Vivas et al., 2011). Primary and secondary antibodies were diluted to a concentration of 1:1,000 in bovine serum albumin (BSA, Sigma) (1% in PBS). To stain intracellular bacteria, cells were permeabilized using Triton X-100 (0.1% in PBS) for 4 min at room temperature. The coverslips were then washed four times with PBS and incubated for 20 min with the primary antibody: polyclonal rabbit anti-*C. striatum* SpaD anti-serum (Beijing Protein Innovation Co., Ltd, China). After washing with PBS for five times, the coverslips were incubated for 20 min with the secondary antibody: YF^®^-488 goat anti-rabbit IgG antibody (H&l; LABLEAD Co., China). Then, fluorescent-labeled phalloidin (Solarbio^®^ Life Science Co., China) was used to stain the cytoskeleton. The coverslips were washed five times with PBS, and DAPI (Vectorlabs, USA) was used to stain the nuclei. After five more washes with PBS, the cells were observed and photographed using AX Confocal Microscope (Nikon, Japan).

### 2.5 Detection of apoptosis and necrosis by flow cytometry

To compare the pathogenic effects of *C. striatum* clinical isolates on human respiratory epithelial cells at different infection stages, the cell viabilities of A549 cells at 2 h postinfection were assessed using flow cytometry assay (Kunyanee et al., 2016). An MOI of 100:1 (bacterium/A549 cell ratio) was used to for 2 h period of infection. A549 cells were seeded into 24-well plates at a density of 2 × 10^5^ cells per well with a bacterial/cellular infection MOI of 100. After 2 h of infection with *C. striatum*, the cells were washed four times with PBS, and adherent cells were treated with 2 min of trypsinization. Thereafter, the cells were washed twice with cold PBS and resuspended in 1 × binding buffer at a concentration of 1 × 10^6^ cells/mL. One hundred microliters of the solution was transferred to a 5-mL culture tube, and 5 μL of FITC Annexin V (BD Biosciences, Cat. No. 556547) and 5 μL of PI were added. The cells were gently vortexed and then incubated for 15 min at room temperature in the dark. 1 × Binding Buffer (400 μL) was added to each tube, and the cells were analyzed by flow cytometry within 1 h. Samples were analyzed using a FACSCalibur system (Becton Dickinson) by gating A549 cells based on forward and side scatter to obtain the percentage of apoptosis or necrosis of cells infected with different *C. striatum* isolates.

### 2.6 Effect of cytochalasin D on actin-dependent internalization of *C. striatum*

To investigate whether actin was involved in the internalization of *C. striatum* into A549 cells, the actin-disrupting agent cytochalasin D was added to prevent the uptake of bacteria by A549 cells (Ramos-Vivas et al., 2011). Generally, A549 cells were preincubated with cytochalasin D (1 μg/mL) for 30 min before infection with *C. striatum* cultures. After 2 h of infection, the unbound bacteria were removed by washing with PBS for five times. Then, 200 μg/mL gentamicin was added to kill extracellular bacteria. The quantity of internalized bacteria was calculated according to the procedure described above. Wells not treated with cytochalasin D (1 μg/mL) were set as the control group.

### 2.7 Statistical analysis

Statistical analysis was performed using SPSS 23.0 and GraphPad 9.5.1, and the results were statistically analyzed using *t*-tests. A *P* value of < 0.05 was considered to indicate statistical significance, and *P* values of < 0.01 were considered to indicate high statistical significance.

## 3 Results

### 3.1 Phylogenetic analysis and virulence genes profiling of *C. striatum* clinical isolates

**Figure 1:**
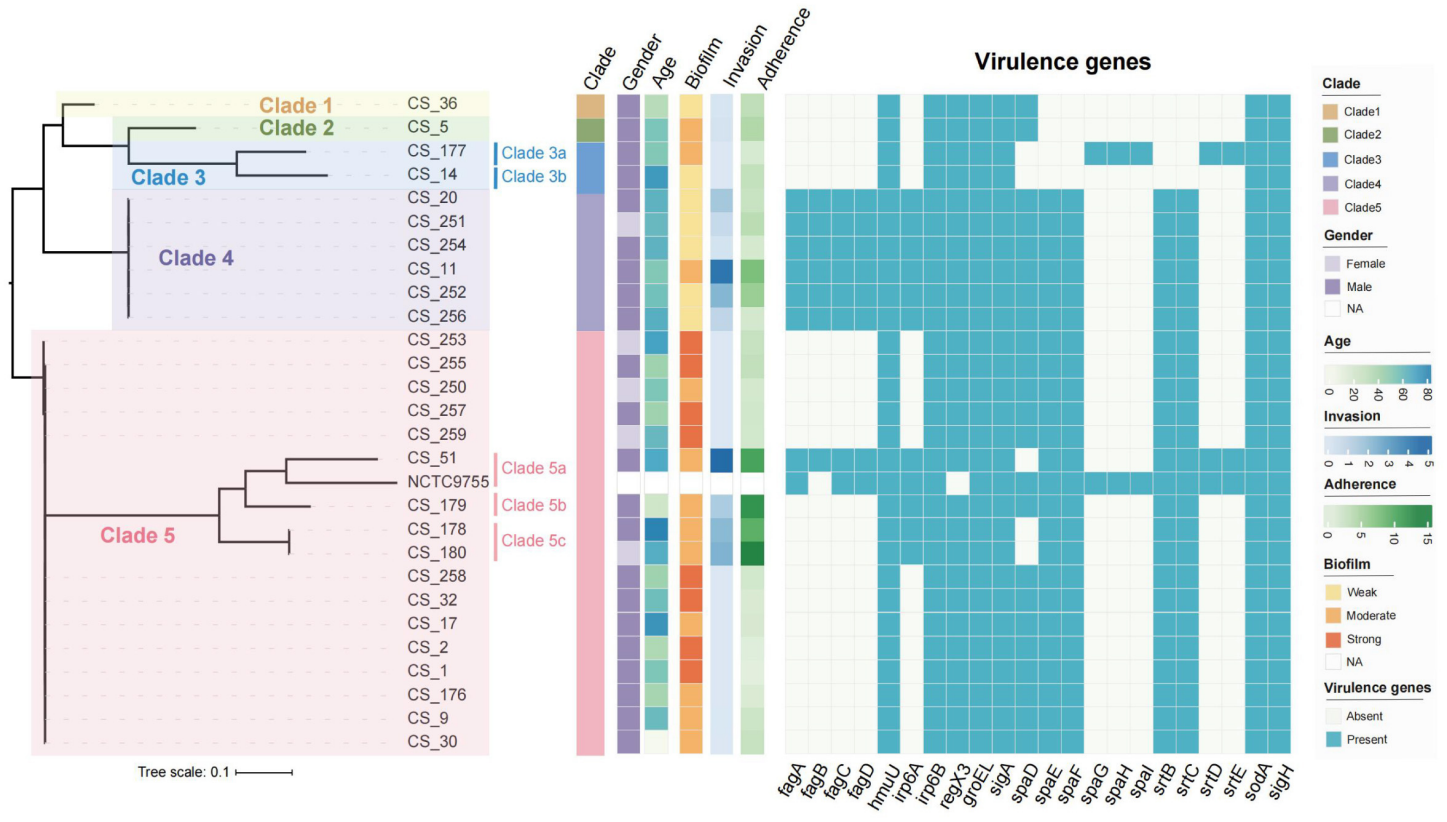
Phylogenetic tree of 27 *C. striatum* clinical isolates. Biofilm formation capabilities were detected and described in one of our previous studies (Wen et al., 2022).

Genomic comparisons revealed 69,056 core genome SNPs among 27 isolates, when the type strain NCTC9755 was used as reference genome. Further phylogenetic analysis revealed that the 27 *C. striatum* isolates were classified into five clades, with clade 5 containing the highest number of isolates (17/27, 62.96%), followed by clades 4, 3, 2, and 1, containing 6, 2, 1, and 1 isolates, respectively. As shown in Figure 1, clade 5 yielded 45,973 SNPs. As the second largest clade, clade 3 yielded 16,310 SNPs. Furthermore, 65 SNPs were identified in clade 4. Three sub-clades (clade 5a, 5b, and 5c) were further identified, all of which were strong or medium biofilm producers. All the strong biofilm producers belonged to clade 5. The detailed genome sequencing data of 27 *C. striatum* clinical isolates were in the National Center for Biotechnology BioProject database (Accession number:SAMN35779365 CS-1, SAMN35779366 CS-36, and PRJNA1106209).

Among the 27 *C. striatum* isolates analyzed in this study, 22 virulence-related genes were identified. These genes participated in several biological functions in *C. striatum*, including adherence and biofilm formation (*spaD, spaE, spaF, spaG, spaH, spaI, srtB, srtC, srtD, strE*), iron uptake and metabolism (*fagA, fagB, fagC, fagD, humU, irp6A, irp6B*), stress responses (*sigA, sigH*), molecular chaperone (*groEL*), intracellular survival (*sodA*), and the two-component regulatory system (*regX3*). The genes *humU, Irp6B, regX3, groEL, sigA, sodA*, and *sigH* were found in all of the tested isolates, whereas *spaE* was found in 85.19% of isolates (23/27), *spaF* in 85.19% (23/27), *srtB* in 85.19% (23/27), *srtC* in 85.19% (23/27), and *spaD* in 81.48% (22/27) (Figure 1). Except NCTC9755, the *spaGHI* and *srtDE* gene clusters were only identified in the *C. striatum* isolate CS-177. Except CS-51 and NCTC9755, *fagABCD* gene clusters were uniquely distributed among the isolates belonging to clade 4. CS-14, deficient in *spaDEF* and *spaGHI* gene clusters, showed the weakest biofilm formation capability; however, CS-5 and CS-36, harboring only the *spaD* subunit, presented with moderate and weak biofilm formation capabilities, respectively.

### 3.2 Adherence and invasion of *C. striatum* in A549 cells

**Figure 2:**
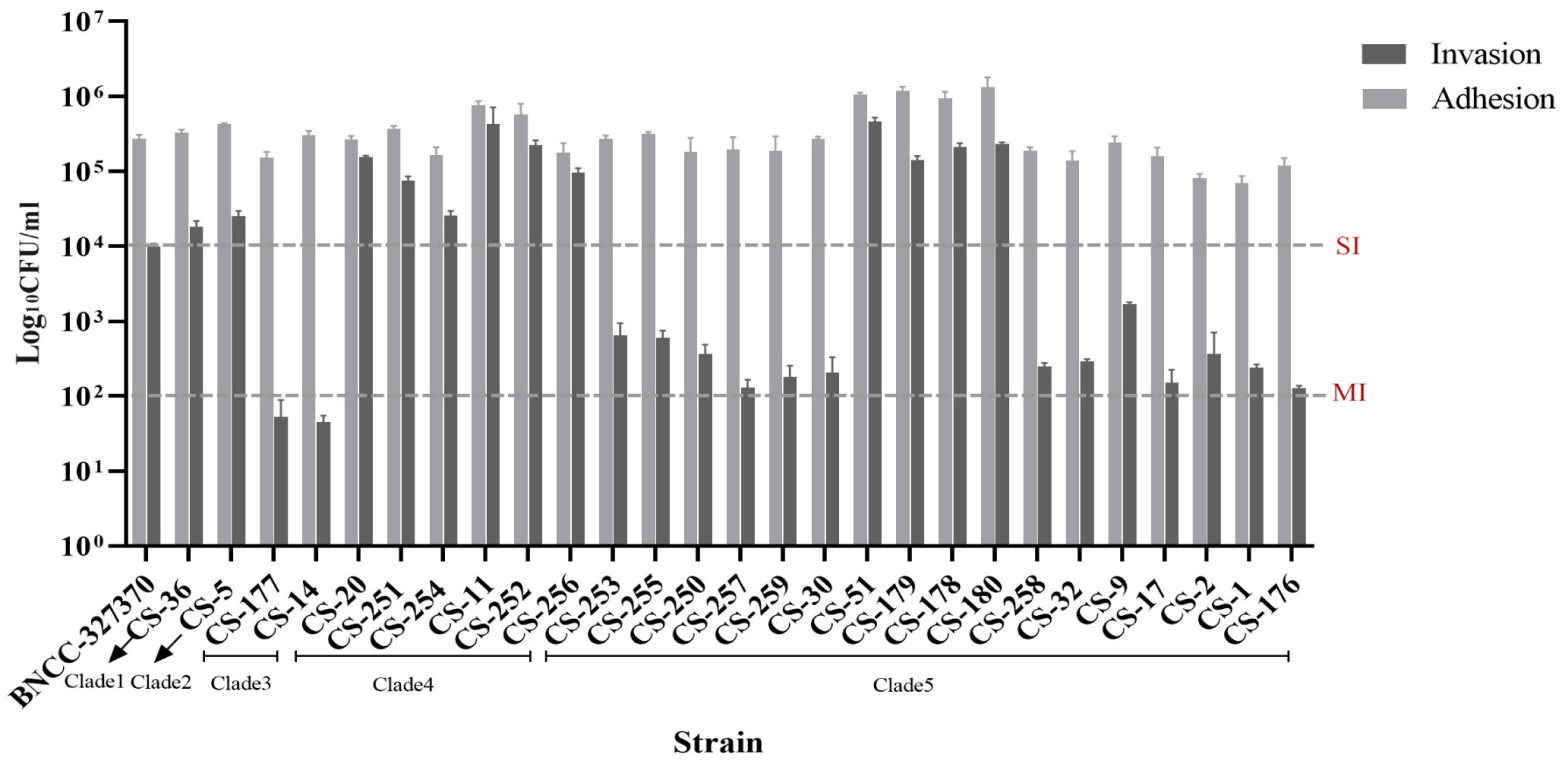
Adherence and invasion capabilities of 27 *C. striatum* isolates in A549 cells. SI, strongly invasive; MI, moderately invasive.

Based on the antimicrobial susceptibility testing results, all of the 27 clinical isolates were susceptible to gentamicin (200 μg/mL; Supplementary Table 2). Also, gentamicin at 200 μg/mL could effectively eliminate extracellular bacteria in the wells with infected A549 cells, when no colony was observed on blood agar plates after 48 h incubation. Antibiotic protection assays revealed that all 27 representative *C. striatum* isolates could adhere and penetrate A549 cells. All isolates showed adherence abilities toward A549 cells, with cellular adherence rates varying widely from 0.694% to 13.305% (Figure 1 and Supplementary Table 1). Meanwhile, the invasion capabilities of the *C. striatum* isolates toward A549 cells also varied greatly among different isolates, ranging from 0.001% to 4.615%. However, no significant correlation between adherence and invasion capability was observed. The invasive capabilities of the *C. striatum* isolates were classified into three categories based on the protocol employed in this study: strongly invasive (SI, number of internalized bacteria >10^4^ Log_10_ CFU/mL after 2 h of infection), moderately invasive (MI, number of internalized bacteria ranging between 10^2^ and 10^4^ Log_10_ CFU/mL after 2 h of infection), and weakly invasive (WI, number of internalized bacteria ≤ 10^2^ Log_10_ CFU/mL after 2 h of infection). Based on this definition, 44.44% (12/27), 48.15% (13/17), and 7.41% (2/27) of the *C. striatum* isolates used in this study were classified as SI, MI, and WI isolates, respectively (Figure 2).

The intracellular invasion capabilities varied greatly among isolates belonging to different clades and within the same clade. Only two WI isolates belonged to clade 3, both of which were *spaD*-negative. All isolates in clade 4 were SI, but 83.3% (5/6) of these isolates were weak biofilm producers, except CS-11. Obvious heterogenicity of invasion capabilities was observed among the isolates belonging to clade 5, where 23.53% (4/17) and 76.47% (13/17) of the isolates showed strong and moderate intracellular invasive capabilities, respectively. All eight isolates with strong biofilm production capabilities belonging to this clade showed moderately invasive capabilities, whereas the four SI isolates were moderate biofilm producers. The *C. striatum* isolate with the most powerful invasion capability (CS-51) belonged to clade 5a and presented medium biofilm production capability. The CS-51 isolate carried 18 virulence genes but was negative for the *spaD* subunit. The results indicated no significant correlation between biofilm production capability and invasion efficiency in the *C. striatum* isolates.

### 3.3 Effect of cytochalasin D on *C. striatum* invasion

**Figure 3:**
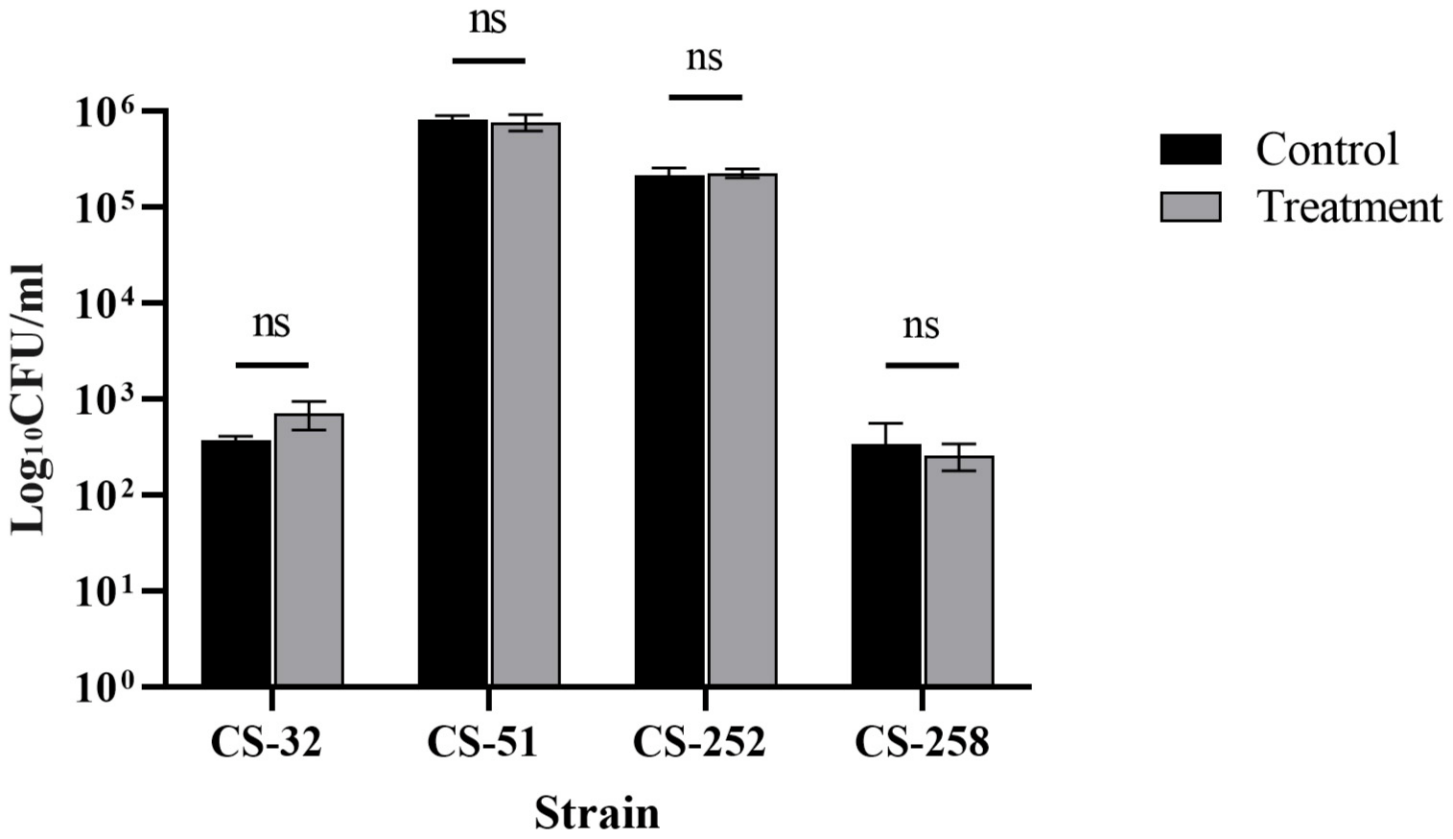
Influence of cytochalasin D on invasion capabilities of *C. striatum* isolates.

Furthermore, no significant effects of cytochalasin D were observed in A549 cells on the invasion efficiencies of four representative *C. striatum* isolates compared with those of the untreated groups (*P* > 0.05) (Figure 3), which indicated a strong cell invasion capability of *C. striatum* and a dispensable role of the host cell cytoskeleton in mediating the process of invasion.

### 3.4 Apoptotic and necrotic effects of *Corynebacterium striatum* on A549 cells

**Figure 4:**
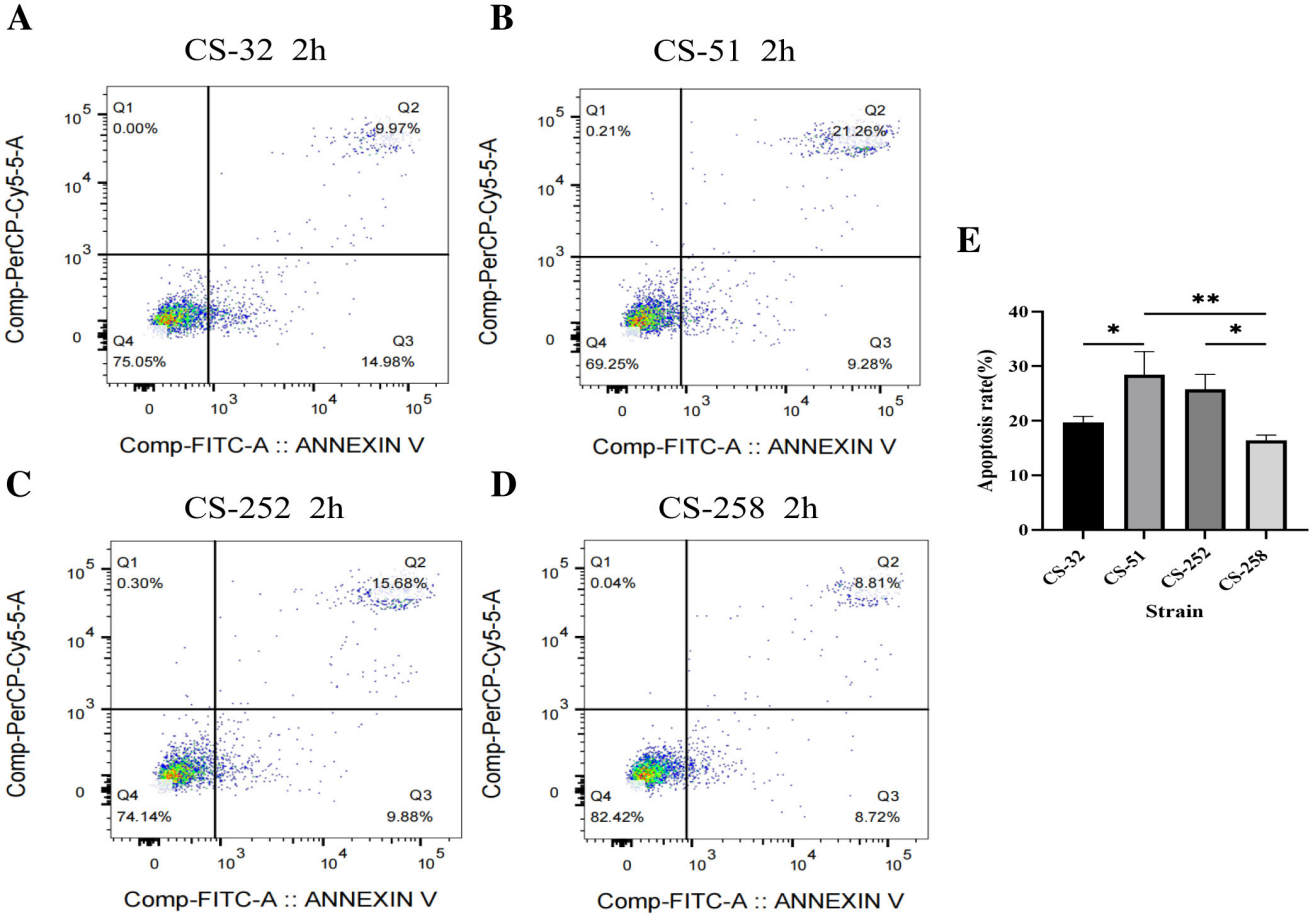
Apoptosis of A549 cells infected with different *C. striatum* isolates. **(A–D)** Apoptotic rates of A549 cells infected with CS-32 (MI), CS-51 (SI), CS-252 (SI), and CS-258 (MI) detected by flow cytometry. **(E)** Quantification of apoptosis rates among infected groups. *P < 0.05; ** *P* < 0.01; ns, no significant difference.

**Figure 5:**
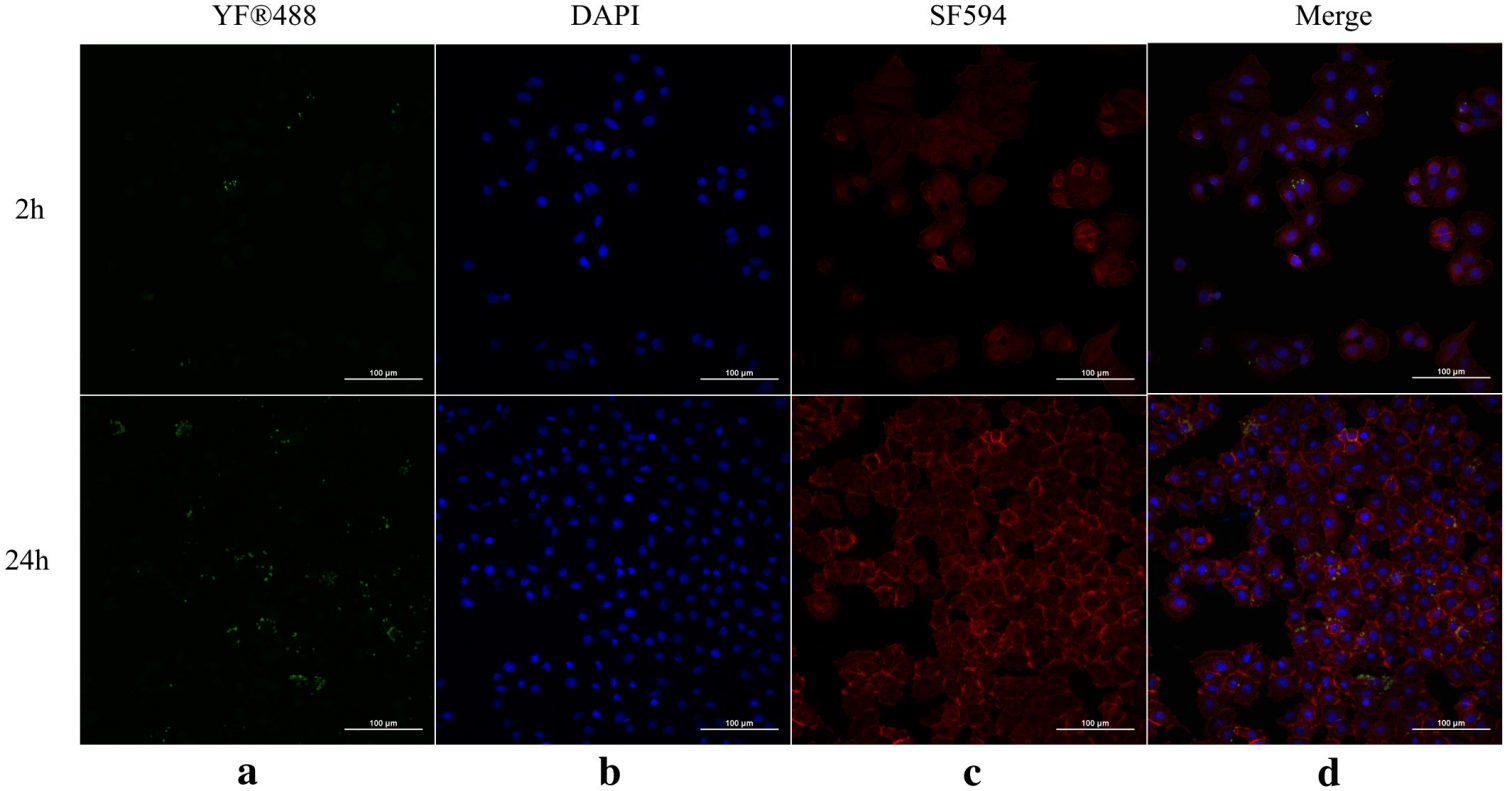
Adherence and invasion of *C. striatum* CS-32 in A549 cells at 2 h and 24 h of infection. **(a)** Bacteria were detected with anti-*C. striatum* SpaD antiserum and YF^®^-488 labeled goat anti-rabbit antibody, and are shown in green. **(b)** DAPI-stained nuclei are shown in blue. **(c)** Phalloidin-labeled cellular F-actin is shown in red. **(d)** Images of extracellular bacteria, nuclei, and cellular F-actin were merged. Micrographs were originally captured at 400× magnification.

The objective of this assay was to analyze and compare possible differences of cytotoxicity by *C. striatum* isolates with different intracellular invasion capabilities. Thus, the four representative isolates (CS-32, CS-51, CS-252, and CS-258) were selected based on their intracellular invasion abilities, when CS-32 and CS-258 were moderately invasive (MI), CS-51 was weakly invasive (WI), and CS-252 was strongly invasive (SI), respectively. Since MI isolates (48.55, 13/27) accounted for the majority among 27 isolates, two MI isolates were selected for this assay. As shown in Figure 4, four representative *C. striatum* isolates could cause obvious apoptosis and necrosis in A549 cells after 2-h of infection. The highest apoptosis rate (30.54%) was observed in A549 cells after 2 h of infection at an MOI of 100:1 by the CS-51 isolate (SI), followed by 25.56% for CS-252 (SI), 24.95% for CS-32 (MI), and 17.53% for CS-258 (MI). Interestingly, heterogeneity of cytotoxity to A549 cells was observed in the isolates belonging to clade 5 (CS-32, CS-51, and CS-258). Generally, the damaging effects of SI isolates were greater than those of WI isolates (*P* < 0.05). Furthermore, *C. striatum* could adhere to and invade A549 cells during 24 h of infection, and proliferate intracellularly, which could be clearly observed using the fluorescent staining technique (Figure 5). As shown in Supplementary Figure 1, no differences in growth rates (ODs) at 2-h were observed among 27 isolates with the same initial concentration. Thus, the invasion abilities and pathogenic effects of different *C. striatum* isolates on A549 cells primarily depend on isolate-specific virulence rather than growth rate.

## 4 Discussion

With increasing reports unveiling infections caused by multidrug resistant *C. striatum* isolates among susceptible inpatients (Silva-Santana et al., 2021; Lee et al., 2022), it is important to explore the pathogenicity and virulence mechanisms of *C. striatum*. Most of the available reports focus only on the biofilm characteristics of *C. striatum*, and few have explored its virulence potential (Souza et al., 2019; Alibi et al., 2021; Wen et al., 2022). Therefore, the detailed mechanisms involved in the virulence of *C. striatum* are poorly understood. In this study, human epithelial A549 cells were employed to explore the intracellular invasion capability of 27 *C. striatum* clinical isolates collected from a tertiary teaching hospital in China. Although the invasive capabilities varied greatly among different *C. striatum* isolates, all isolates were able to penetrate A549 cells after 2 h of infection. To the best of our knowledge, this is the first report to reveal the intracellular invasion capabilities of *C. striatum*. Among *Corynebacterium, C. diphtheriae* and *C. ulcerans* have been confirmed to be invasive (Hacker et al., 2015; Peixoto et al., 2017), with intracellular invasion rates comparable to those of the *C. striatum* isolates detected in this study (range, 0.006%−5.8%). Because great variations in invasion capabilities toward A549 cells among different *C. striatum* isolates were observed in this study, we subjectively classified the invasion capabilities of *C. striatum* isolates based on the *in vitro* infection protocol used in this study. Importantly, the intracellular bacterial densities of 29.62% (8/27) of *C. striatum* isolates reached Log_10_10^5^CFU/mL after 2 h of infection, which was significantly higher than that reported in a previous study (Mangutov et al., 2022). Considering the heterogenicity of the bacterial strains detected and the cell lines selected in different reports, it is inappropriate to compare the actual deviations of invasion capabilities from different origins. Thus, it is essential to establish a standardized protocol for evaluating the *in vitro* invasion capabilities of bacterial pathogens in different laboratories, the key points of which should include cell lines, infection MOI, infection times, and conditions.

The decision to conduct the cellular invasion experiment for 2 h was based on two considerations. First, 2 h is a standard period used in several previous studies to assess the cellular invasion efficiencies of well-known bacterial pathogens, including *Staphylococcus aureus* and *Rhodococcus equi* (Ramos-Vivas et al., 2011; Truong-Bolduc et al., 2015). Second, no differences in growth rates at 2-h of culture *in vitro* were observed among 27 *C. striatum* clinical isolates. Considering these two aspects, 2 h was selected for the cellular invasion experiment in this study.

Some potential indications should be mentioned regarding the fates of the internalized bacteria and the infected host cells. Considering the higher relative abundance of *C. striatum* within the microenvironment of the upper respiratory tract of susceptible patients (Santos et al., 2015; Wang et al., 2016), it is reasonable to speculate that intracellular invasion, persistence, and/or proliferation by *C. striatum* in human respiratory epithelial cells is a common phenomenon. Second, serious damage to A549 cells infected by *C. striatum* was observed after 2 h of incubation with almost all isolates detected, and both apoptosis and necrosis were detected. However, the apoptosis rates of the infected A549 cells after 2 h of infection were relatively lower than those caused by *C. diphtheriae, C. ulcerans* (Wang et al., 2016; Weerasekera et al., 2019), or *Pseudomonas aeruginosa* (Siddiqui et al., 2015). Thus, internalized bacteria within epithelial cells may act as reservoirs for long-lasting local colonization, long-distance spread, or progression of infection caused by *C. striatum*. Third, the internalization process seemed to be mainly accomplished due to active invasion by *C. striatum*, because the addition of cytochalasin D had no influence on the internalization of *C. striatum*, which implies a strong intracellular invasion capability of this pathogen. In addition, significant isolate heterogeneity for intracellular invasion and cytotoxicity capability was observed, where representative SI isolates could lead to significant pathogenic effects in A549 cells. However, the mechanism underlying the intracellular invasion and cytotoxicity of *C. striatum* could not be determined in the present study.

In this study, we attempted to establish the relationships between the carriage of adherence-related genes, biofilm formation, and intracellular invasion potentials in *C. striatum*. No definite correlation between biofilm production capabilities and virulence potential has yet been identified. Adherence is a key determinant in biofilm formation, and several adherence-related genes have been confirmed to be related to biofilm formation in *C. striatum*, including *spaDEF* gene clusters as well as its related *sortase*, s*rtB* and s*trtC* (Gaspar and Ton-That, 2006). We found that the prevalence of s*rtC*, s*rtB*, and *spaDEF* was significantly higher than that reported in a previous study (Qiu et al., 2023). SrtB and SrtC are sortases essential for the formation of *spaDEF*-encoded pili in *C. diphtheria* (Gaspar and Ton-That, 2006). Although adherence is a prerequisite for bacterial internalization, specific adherence-related genes, such as the *spaDEF* cluster, are not essential for intracellular invasion by *C. striatum*. The *spaDEF* genes were previously reported to be not only an important gene cluster participating in the formation of pili and the mediation of adherence of *C. striatum* to human epithelial cells but also an important factor correlated with the pathogenicity of *C. striatum* (Alibi et al., 2021). As shown in Supplementary Table 1, 27 isolates exhibited diverse adherence capabilities to A549 cells, ranging from 0.694% to 13.305%, as well as those for invasion capability. In general, the correlation between adherence and invasion capabilities for the isolates belonged to clade 5 was not definite. However, the consistency was actually observed among the four strongly invasive isolates (SI), namely CS-51, CS-179, CS-178 and CS-180. Upon analysis of the distribution of 22 predicted virulence genes among the four isolates, the only difference is *spaD* gene, which was absent in three isolates (CS-51, CS-178 and CS-180). Nevertheless, limited by the existing knowledge about the relationship between carriage of specific virulence genes and the adherence or invasion capability for some common bacterial pathogens, including *S. aureus* (Song et al., 2025), it is not enough to speculate this consistency. Other potential mechanisms independent of specific genes profiling needs further investigation, which may contribute to elevated adherence and invasion simultaneously. Interestingly, only two isolates (CS-177 and CS-14) lacked *spaDEF* gene clusters. In addition, *spaGHI* gene clusters, which were previously identified as gene clusters encoding a distinct pilus organelle in *C. diphtheria* (Swierczynski and Ton-That, 2006), were newly reported in this study. Only one isolate (CS-177) carried this gene cluster but lacked the *spaDEF* gene cluster and presented with a weak intracellular invasion capability and undiminished adherence ability. Furthermore, although all eight strong biofilm producers detected in this study carried *spaDEF* gene clusters, none were SI. In contrast, 58.33% (7/12) of the moderate biofilm producers carrying *spaDEF* gene clusters had attenuated invasion capabilities. Therefore, the actual roles of *spaDEF* or *spaGHI* in the mediation of adherence, biofilm formation and intracellular invasion in *C. striatum* is likely to be controlled by a complicated mechanism. These results indicate no definite correlation between biofilm-forming ability and intracellular invasion capacity in *C. striatum*. This discrepancy may be explained well based on existing knowledge about another well-known bacterial pathogen, namely *Staphylococcus aureus*. Initial attachment or adherence is a fundamental and critical step for further biofilm formation and intracellular invasion in *S. aureus*, which is mediated by multiple staphylococcal adhesins, such as *fnbA, fnbB, clfA, sdrD, sdrE* (Idrees et al., 2021). However, the ability of *S. aureus* to adhere to host cells was not associated with a single gene or expression pattern of characteristic adhesion proteins, and was also not correlated with invasion. Also, the wide variation in expression levels of adherence genes was observed among different *S. aureus* clinical isolates (Song et al., 2025). Regarding to *C. striatim*, adherence related genes (such as *spaDEF*) also serve as important candidates in mediating adherence to host cells and early stage of biofilm formation. However, the roles of other factors independent of surface architecture can't be accurately evaluated based on existing information about *C. striatum*, which may play even more important roles in maturity of biofilm formation and intracellular invasion or persistence. Thus, it is reasonable to conclude that a obvious correlation between biofilm formation capability and intracellular invasion ability of *C. striatum* can't be established.

To further ascertain the key genetic determinants that contribute to intracellular invasion and survival of *C. striatum* in host cells, whole-genome sequencing was employed in this study. The highly expressed virulence-related genes identified in this study deserves further attention. The *fagABCD* gene clusters involved in iron uptake were predominantly distributed among the isolates belonging to clade 4, which was previously identified among *C. pseudotubercubosis* (Billington et al., 2002). As a common two-component transduction system, *regX3* was expressed among all strains detected in this study, *which* was previously reported to be participated in regulation of growth, butenyl-spinosyn biosynthesis and adaptation to physiologically relevant conditions in *Saccharopolyspora pogona* (Rang et al., 2020) and *Mycobacterium tuberculosis* (Rifat and Karakousis, 2014). The *sodA* gene was found in all the strains detected in this study and was found to be an important virulence factor in mediating intracellular survival in *M. tuberculosis* (Edwards et al., 2001). The actual roles and mechanisms of these virulence genes in mediating the mechanisms of intracellular invasion and cytotoxicity of *C. striatum* in host cells require further investigation. Limited by our knowledge of the pathogenesis in *C. striatum*, further data mining of the whole genome sequencing information of the 27 *C. striatum* clinical strains didn't fall the scope of this study, which calls for further exploration.

## 5 Conclusions

In conclusion, this study is the first to report robust intracellular invasion and cytotoxicity capabilities of *C. striatum* in human epithelial cells, highlighting the important virulence feature in contributing to intracellular survival and long-distance dissemination. Given *C. striatum*'s high prevalence in clinical infectious samples, robust biofilm formation capabilities, and multidrug resistance, the pathogenic characteristics revealed in this study underscore its clinical importance, particularly for isolates with high invasive potential. Further exploration of *C. striatum*'s pathogenic mechanisms is urgently required, as our understanding of this pathogen remains limited.

## Data Availability

The datasets presented in this study can be found in online repository and Supplementary material. The names of the repository and accession numbers can be found in the article, as described in the results section 3.1.

## Supplementary material

Supplementary Table 1 Adherence and invasion rates of 27 *C. striatum* isolates.

Supplementary Table 2 Results of gentamicin susceptibility test of 27 *C. striatum* isolates.

Supplementary Figure 1 Growth curves of 27 *C. striatum* clinical isolates.

Supplementary Data 1 Absorbance values (OD620_nm_) of 27 *C. striatum* isolates over a 24-h period in a liquid shaking culture.
