# Supplementary material for: Intracellular invasion potential and pathogenic effects of Corynebacterium striatum clinical isolates in human airway epithelial cells
Source: Front Microbiol. 2025 Jul 28;16:1647771. doi: 10.3389/fmicb.2025.1647771 (PMC12336147; doi:10.3389/fmicb.2025.1647771)
Supplement: Supplementary Table 1 — Adherence and invasion rates of 27 C. striatum isolates. [file Table_1.docx]

Supplementary table 1 Adherence and invasion rates of 27 *C.striatum* strains

| Strain | Adherence rate (%) | Invasion rate (%) |
| --- | --- | --- |
| BNCC-327370 | 2.698±0.385 | 0.099±0.009 |
| CS-36 | 3.307±0.277 | 0.183±0.035 |
| CS-5 | 4.292±0.103 | 0.253±0.044 |
| CS-177 | 1.530±0.291 | 0.001±0.000 |
| CS-14 | 3.023±0.445 | 0.000±0.000 |
| CS-20 | 2.644±0.323 | 1.569±0.050 |
| CS-251 | 3.655±0.386 | 0.752±0.099 |
| CS-254 | 1.663±0.437 | 0.258±0.038 |
| CS-11 | 7.600±1.106 | 4.263±2.886 |
| CS-252 | 5.758±2.198 | 2.220±0.369 |
| CS-256 | 1.772±0.599 | 0.962±0.134 |
| CS-253 | 2.702±0.281 | 0.007±0.003 |
| CS-255 | 3.168±0.192 | 0.006±0.002 |
| CS-250 | 1.815±0.998 | 0.004±0.001 |
| CS-257 | 1.969±0.886 | 0.001±0.000 |
| CS-259 | 1.891±1.040 | 0.002±0.001 |
| CS-30 | 2.703±0.196 | 0.002±0.001 |
| CS-51 | 10.480±0.798 | 4.615±0.599 |
| CS-179 | 11.880±1.557 | 1.411±0.201 |
| CS-178 | 9.333±2.233 | 2.118±0.256 |
| CS-180 | 13.305±4.624 | 2.342±0.108 |
| CS-258 | 1.885±0.217 | 0.003±0.000 |
| CS-32 | 1.384±0.481 | 0.003±0.000 |
| CS-9 | 2.395±0.537 | 0.017±0.001 |
| CS-17 | 1.600±0.479 | 0.002±0.001 |
| CS-2 | 0.805±0.118 | 0.004±0.003 |
| CS-1 | 0.694±0.168 | 0.002±0.000 |
| CS-176 | 1.187±0.312 | 0.001±0.000 |
