# Supplementary material for: Intracellular invasion potential and pathogenic effects of Corynebacterium striatum clinical isolates in human airway epithelial cells
Source: Front Microbiol. 2025 Jul 28;16:1647771. doi: 10.3389/fmicb.2025.1647771 (PMC12336147; doi:10.3389/fmicb.2025.1647771)
Supplement: Supplementary Table 2 — Results of gentamicin susceptibility test of 27 C. striatum isolates. [file Table_2.docx]

Supplementary Table 2 Results of gentamicin susceptibility test of 27 *C.striatum* isolates

| **Isolates** | **MIC (μg/mL)** | **Antimicrobial susceptibility** |
| --- | --- | --- |
| CS-36 | ≤4 | Sensitive |
| CS-5 | ≤4 | Sensitive |
| CS-177 | ≤4 | Sensitive |
| CS-14 | ≤4 | Sensitive |
| CS-20 | ≤4 | Sensitive |
| CS-251 | ≤4 | Sensitive |
| CS-254 | ≤4 | Sensitive |
| CS-11 | ≤4 | Sensitive |
| CS-252 | ≤4 | Sensitive |
| CS-256 | ≤4 | Sensitive |
| CS-253 | ≤4 | Sensitive |
| CS-255 | ≤4 | Sensitive |
| CS-250 | ≤4 | Sensitive |
| CS-257 | ≤4 | Sensitive |
| CS-259 | ≥16 | Resistant |
| CS-30 | ≤4 | Sensitive |
| CS-51 | ≥16 | Resistant |
| CS-179 | ≤4 | Sensitive |
| CS-178 | 8 | Intermediate |
| CS-180 | 8 | Intermediate |
| CS-258 | ≤4 | Sensitive |
| CS-32 | ≤4 | Sensitive |
| CS-9 | ≤4 | Sensitive |
| CS-17 | ≤4 | Sensitive |
| CS-2 | ≤4 | Sensitive |
| CS-1 | ≤4 | Sensitive |
| CS-176 | ≤4 | Sensitive |
