# Supplementary figures and images for: Intracellular invasion potential and pathogenic effects of Corynebacterium striatum clinical isolates in human airway epithelial cells
Source: Front Microbiol. 2025 Jul 28;16:1647771. doi: 10.3389/fmicb.2025.1647771 (PMC12336147; doi:10.3389/fmicb.2025.1647771)

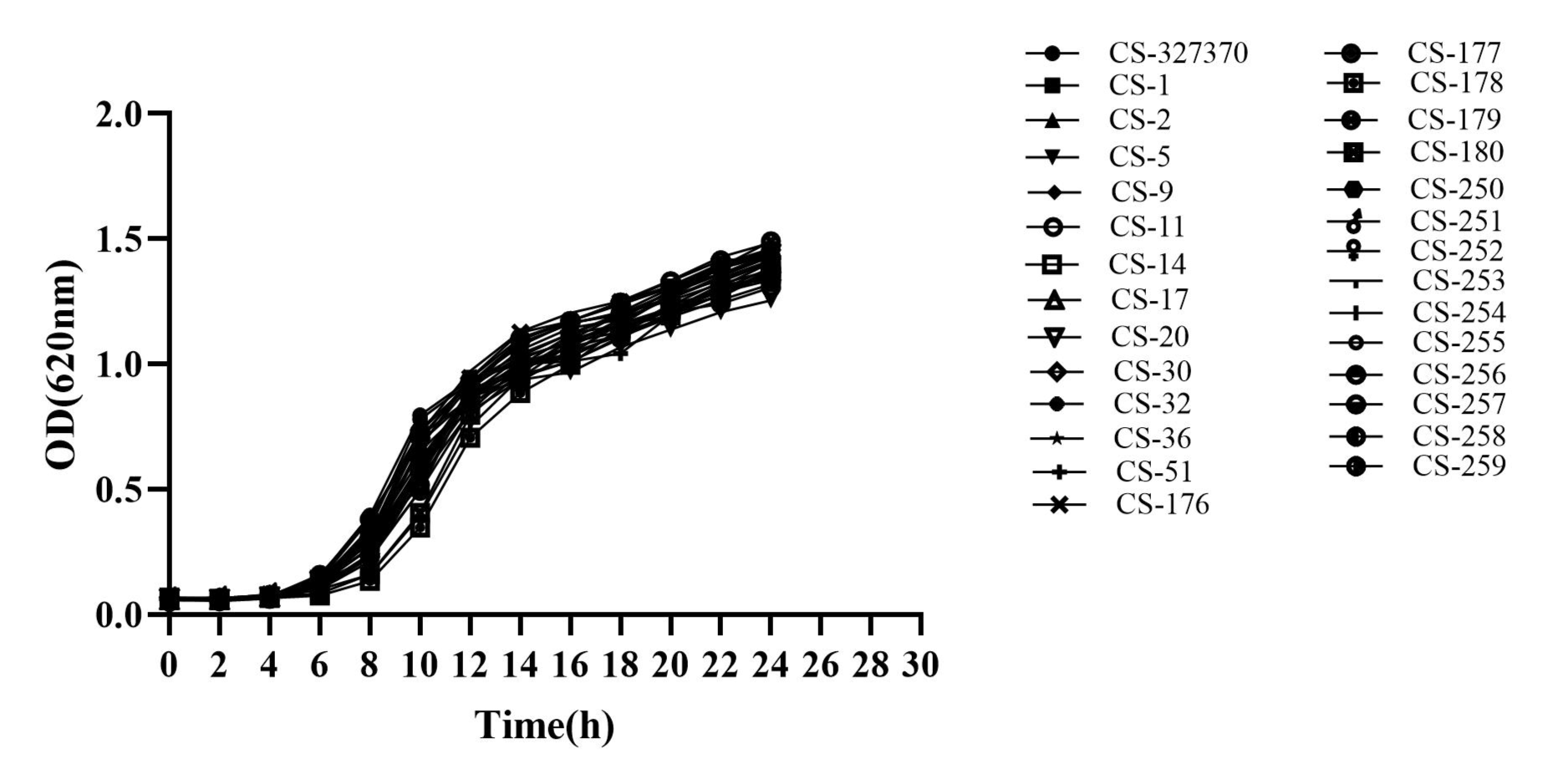

Supplement: Supplementary Figure 1 — Growth curves of 27 C. striatum clinical isolates. [file Image_1.tif]
